# Multi-Objective Optimization of Surface Roughness, Cutting Force, and Temperature in Ultrasonic-Vibration-Assisted Milling of Titanium Alloy

**DOI:** 10.3390/mi16080936

**Published:** 2025-08-14

**Authors:** Gaofeng Hu, Yanjie Lu, Shengming Zhou, Xin He, Fenghui Zhang, Pengchao Zhu, Mingshang Wang, Taowei Tan, Guangjun Chen

**Affiliations:** 1 College of Mechanical Engineering, Tianjin University of Technology and Education, Tianjin 300222, China; 17860385863@163.com (Y.L.); 0322231061@tute.edu.cn (S.Z.); zfh0476@126.com (F.Z.); 17633280330@163.com (P.Z.); 15680998938@163.com (M.W.); otantaoweio@163.com (T.T.); chenguangjun@126.com (G.C.); 2 Tianjin Key Laboratory of High Performance Manufacturing Technology & Equipment, Tianjin 300222, China; 3 College of Mechanical Engineering, Tianjin University, Tianjin 300222, China; 4 Engineering Training Center, Tianjin University of Technology and Education, Tianjin 300222, China; kernel139@163.com

**Keywords:** ultrasonic-vibration-assisted milling, multi-objective optimization, surface roughness, cutting force, cutting temperature

## Abstract

Titanium alloys (Ti-6Al-4V) are widely used in the aerospace field. However, as a typical difficult-to-machine material, titanium alloys have a low thermal conductivity, a high chemical activity, and a significant adiabatic shear effect. In conventional milling (CM), the temperature in the cutting zone rises sharply, leading to tool adhesion, rapid wear, and damage to the workpiece surface. This article systematically investigated the influence of process parameters on the surface roughness, cutting force, and cutting temperature in the ultrasonic-vibration-assisted milling (UAM) process of titanium alloys, based on which multi-objective optimization process of the milling process parameters was conducted, by utilizing the grey relational analysis method. An orthogonal experiment with four factors and four levels was conducted. The effects of various process parameters on the surface roughness, cutting force, and cutting temperature were systematically analyzed for both UAM and CM. The grey relational analysis method was employed to transform the optimization problem of multiple process target parameters into a single-objective grey relational degree optimization problem. The optimized parameter combination was as follows: an ultrasonic amplitude of 6 μm, a spindle speed of 6000 rpm, a cutting depth of 0.20 mm, and a feed rate of 200 mm/min. The experimental results indicated that the surface roughness Sa was 0.268 μm, the cutting temperature was 255.39 °C, the cutting force in the X direction (FX) was 5.2 N, the cutting force in the Y direction (FY) was 7.9 N, and the cutting force in the Z direction (FZ) was 6.4 N. The optimization scheme significantly improved the machining quality and reduced both the cutting forces and the cutting temperature.

## 1. Introduction

Titanium alloys are widely used in aerospace, bioengineering, and other fields because of their excellent characteristics, such as a high strength and hardness, strong corrosion resistance, and an excellent thermal resistance to temperature changes [1]. However, titanium alloys have a small elastic modulus, poor thermal conductivity, strong chemical activity, and a poor machining performance, which largely limit their application [2]. Therefore, how to effectively reduce the processing cost of titanium alloys and significantly improve their processing efficiency and processing quality has become a critical issue that needs to be addressed urgently at present [3]. With the continuous development of ultrasonic machining technology, the ultrasonic-assisted machining of titanium alloys has several advantages. It can reduce the stress and workpiece deformation during the machining process, decrease the cutting heat, and provide good vibration-damping and chatter-suppression capabilities. These advantages can effectively improve machining deformation and enhance the machining accuracy and surface quality.

In the field of ultrasonic vibration machining technology for difficult-to-machine materials, several scholars have conducted relevant experimental studies. Celaya et al. studied the advantages and disadvantages of ultrasonic-assisted turning, with a focus on how the effect of ultrasonic vibrations on surface roughness varies with the cutting conditions [4]. In addition, they explored the application of modulation-assisted machining and ultrasonic-assisted machining in mechanical processing [5]. Chern et al. [6] carried out micro-milling experiments on Al6061 using a two-dimensional ultrasonic vibration device. The results indicated that ultrasonic-assisted milling could improve the dimensional accuracy of the grooves and enhance the surface roughness. Ding et al. [7] conducted experiments on the ultrasonic-assisted milling of tool steel and found that the surface roughness gradually decreased with an increase in the ultrasonic amplitude and vibration frequency. Chen et al. [8] performed comparative experiments between conventional milling and ultrasonic-vibration-assisted milling on Ti-6Al-4V titanium alloy workpieces at spindle speeds ranging from 47 to 75 m/min. The results showed that ultrasonic-vibration-assisted milling significantly improved the machining quality, resulting in a lower surface roughness. Hu et al. [9] investigated the surface integrity of nickel-based superalloys under ultrasonic elliptical vibration cutting conditions. The influence of different process parameters on the surface roughness, residual stress, and microhardness of workpieces was presented. Iji Shamoto et al. [10] utilized three-dimensional ultrasonic vibration cutting for diamond. Their experimental results demonstrated that three-dimensional ultrasonic vibration significantly improved the surface quality of the diamond and the tool life while also optimizing the micro-machining technology and enhancing the machining efficiency. Tong et al. [11] found that, during ultrasonic longitudinal vibration machining, the flank face of the tool impacts the machined surface, thereby reducing the tool life. In contrast, ultrasonic longitudinal–torsional composite machining could improve the tool life. Suárez A et al. [12] demonstrated that the fatigue life of nickel-based superalloy workpieces machined by ultrasonic-assisted milling was 14.74% higher than that of workpieces machined by conventional milling. Zhao Bo [13] investigated the influence of the ultrasonic vibration direction on the milling characteristics of titanium alloys and found that, compared with horizontal ultrasonic vibration, axial ultrasonic vibration was more effective at reducing the milling force and tool wear. Ahmar Khan [14] conducted a study on the material removal mechanism and surface generation of an intermetallic Ti2AlNb compound alloy during ultrasonic vibration machining, and discovered that ultrasonic vibration machining can effectively enhance the machining quality of a Ti2AlNb alloy. Zhang [15] designed an ultrasonic vibration tool holder and carried out cutting experiments. Their results showed that the ultrasonic vibration milling of a TC4 titanium alloy exhibited a better performance in terms of the milling force and surface quality.

In the field of milling process optimization, several scholars have conducted relevant studies. Gonzalez proposed a new cutting force prediction model and verified its effectiveness through the experimental testing of representative test components [16]. Kang Chunhui et al. [17] employed the orthogonal test and grey relational analysis method to study the milling process of a Ti-6Al-4V titanium alloy. By optimizing the cutting parameters, the surface chip adhesion rate and surface roughness were reduced. Lin et al. [18] performed the multi-objective optimization of the titanium alloy milling process based on the RBFNN-MOPSO algorithm. The energy consumption and combined bending moment were effectively reduced by considering both the energy efficiency and the tool wear, and the machining efficiency was improved. Liu et al. [19] used a combination of infrared thermal imaging and a finite element simulation to study the temperature field during titanium alloy milling. Based on the whale optimization algorithm, they conducted multi-objective parameter optimization, achieving a dual improvement in machining efficiency and quality. Guo et al. [20] established a milling force model for TC4 titanium alloys and achieved the multi-objective optimization of the milling force and material removal rate based on the Pareto simulated annealing algorithm. Yang et al. [21] addressed the issue of high tool costs for titanium alloy structural aviation components by constructing an evaluation model based on manufacturing costs. Through orthogonal tests, they optimized the milling process parameters, achieving a good match between the process parameters and new materials and tools. Shao et al. [22] proposed an improved particle swarm optimization algorithm, constructed a multi-objective optimization model, and determined the optimal milling parameters, thereby increasing the material removal rate and machining efficiency. Currently, research on ultrasonic-vibration-assisted milling technology in titanium alloy machining mainly focuses on improving the surface roughness or decreasing the cutting forces. However, comprehensive optimization studies for multiple objectives, such as the surface roughness, cutting forces, and temperature, are scarce. The existing studies have tended to focus on the impact of individual factors, lacking a comprehensive consideration of the combined effects of different processing parameters on the surface quality and mechanical properties. Therefore, this study conducted an in-depth exploration of the process parameters for the ultrasonic-vibration-assisted milling of titanium alloys using a multi-objective optimization method, aiming to provide theoretical support for the efficient machining of titanium alloys.

This article systematically studied the influence of the process parameters of ultrasonic-vibration-assisted milling on the surface roughness, cutting force, and cutting temperature in TC4 titanium alloy machining. The grey correlation analysis method was used to optimize key process parameters with multiple objectives. In addition, this paper compared and analyzed the differences between the conventional cutting and ultrasonic vibration cutting of titanium alloys during the machining process, providing a new approach for achieving the high-quality and efficient machining of titanium alloys. The remainder of this paper is organized as follows: the basic principles and experimental design of ultrasonic vibration milling are described in Section 2; the experimental results for the cutting force, temperature, and surface roughness are analyzed, which revealed the influence of the process parameters in ultrasonic-vibration-assisted milling on TC4 titanium alloy machining; the grey relational analysis method is utilized to perform multi-objective optimization on key process parameters; and the findings of this paper are summarized in Section 5.

## 2. The Mechanism of UAM and Experimental Equipment

### 2.1. The Mechanism of UAM

**Figure 1:**
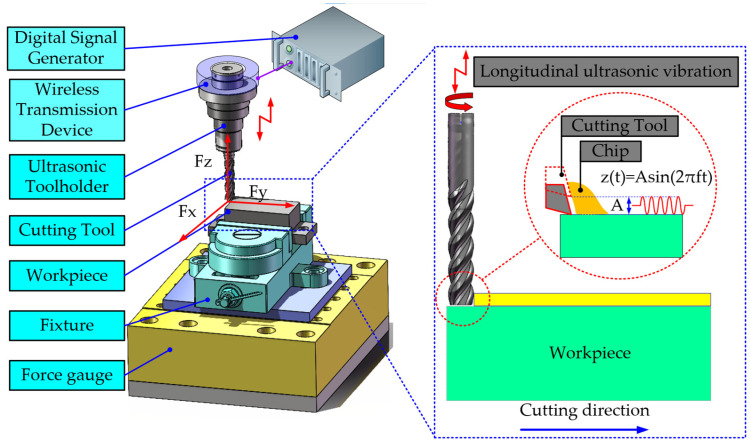
Schematic diagram of ultrasonic vibration milling.

Ultrasonic-assisted milling is a composite machining technology that combines ultrasonic frequency vibration with traditional rotary milling motion [23], as shown in Figure 1. During the machining process, the tool not only performs conventional rotational and feed motions, but also undergoes high-frequency and micro-amplitude vibration along a specific direction (Z direction) under the action of an ultrasonic transducer. This high-frequency vibration causes periodic changes in the contact state between the tool and the workpiece, thereby altering physical phenomena such as the mechanics and thermodynamics during the cutting process. It is particularly suitable for the precision machining of high-strength and low-thermal-conductivity materials like titanium alloys [24,25,26].

Assume that the amplitude of the sinusoidal vibration used for milling is A, with the unit of μm, and the frequency is f, with the unit of Hz. Then, at any time t, the axial displacement z(t) of the tool can be expressed as follows:

(1) $$z(t)=Asin\left(2\pi ft\right)$$

Therefore, during the machining process, the relationship between the axial velocity v(t) of the tool and its displacement can be expressed by the following equation:

(2) $$v(t)=2\pi Afcos\left(2\pi ft\right)$$

Similarly, the relationship between the motion trajectory of the n-th cutting edge of the tool and time t can be represented by the following equation:

(3) $$\left\{\begin{array}{l} x_{n}=vt+rsin(\omega t-n\Phi )\\ y_{n}=rcos(\omega t-n\Phi )\\ z_{n}=Asin\left(2\pi ft+\varphi \right) \end{array}\right.$$

where x_n_, y_n_, and z_n_ represent the coordinate positions of the n-th tool edge during the milling process; r denotes the radius of the milling cutter; v represents the feed rate; Φ indicates the tooth angle; ω signifies the rotational angular frequency of the tool; and φ represents the initial phase angle of the vibration signal.

### 2.2. Experimental Equipment and Materials

**Figure 2:**
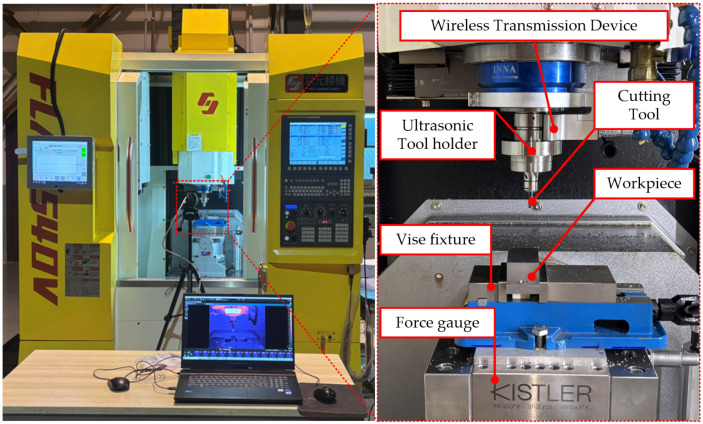
Experimental equipment of ultrasonic vibration milling.

**Figure 3:**
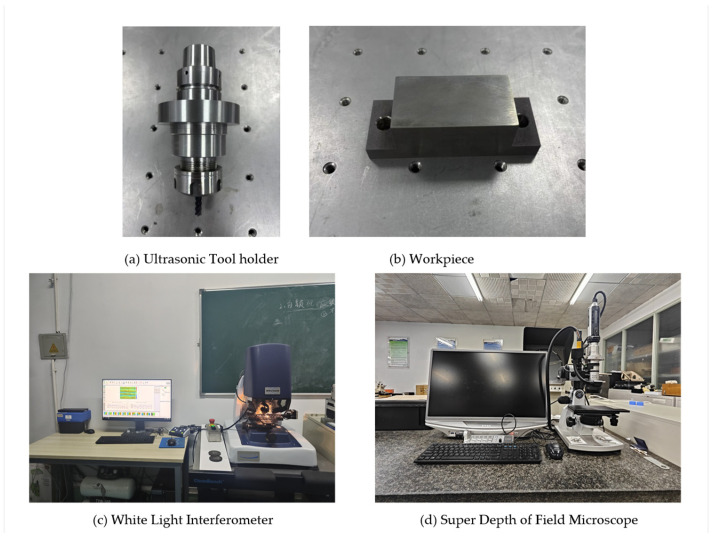
Ultrasonic tool holder and workpiece. Measurement equipment used in the experiment.

The experiment was conducted on the FLM540V precision machining center, with XYZ axis strokes of 400 mm, 400 mm, and 300 mm and a maximum spindle speed of 9000 rpm, manufactured by Kunshan Jingyuan Deng Precision Machinery Co., Ltd. (Kunshan, China), as shown in Figure 2. During the cutting process, a longitudinal ultrasonic vibration cutting device, as shown in Figure 3a, was used, which was installed on the spindle of the machining center. The resonance frequency range was 30 ± 0.75 kHz, and the amplitude was adjustable within 4–10 μm, provided by Qingding Company (Shenzhen, China). The high-frequency vibration signal drove the piezoelectric actuator via a wireless transmission device, and the piezoelectric actuator converted the high-frequency signal into axial vibration of the tool. The vibration waveform was monitored in real time by a digital signal generator. The titanium alloy workpiece was fixed by a vise fixture. A comparative experiment between ultrasonic-assisted milling (UAM) and conventional milling (CM) under different cutting parameters was conducted. The three-dimensional cutting forces were synchronously collected using a three-axis piezoelectric dynamometer (Kistler 9139AA, Kristler, Winterthuru, Switzerland). The direction of the cutting force is shown in Figure 1. During the machining process, the temperature field distribution in the cutting area was synchronously recorded in real time by an infrared thermal imaging camera (FLIR A655sc, FLIR Systems, Wilsonvilleu, OR, USA).

The workpiece material used in this milling experiment was a TC4 titanium alloy (Ti-6Al-4V), with a workpiece size of 36 mm × 66 mm × 20 mm. The chemical composition and key mechanical properties of the TC4 titanium alloy material are listed in Table 1 and Table 2, respectively [27].

The selected tool was a GM-series cemented carbide end mill with a tungsten steel substrate. It was equipped with four cutting edges and had a helix angle of 55°. The tool’s surface was coated with a TiAlN layer through the physical vapor-deposition (PVD) process. The coating thickness ranged from 3 to 5 μm, and its microhardness was not less than 3200 HV.

## 3. Experimental Arrangement and Results Analysis

### 3.1. Experimental Arrangement and Results

In this study, the spindle speed, cutting depth, ultrasonic amplitude, and feed rate were selected as variables, and a four-factor, four-level orthogonal experimental design was developed. Each milling length was 30 mm, and all were full tooth-milling operations. Subsequently, experiments on the ultrasonic vibration milling of titanium alloys were carried out. The data collected in the experiment were based on the average values of each level of the process parameters obtained from the orthogonal experimental design, reflecting the influence trend of each factor on the surface roughness, cutting temperature, and cutting force. The specific parameter settings of the orthogonal experiments are detailed in Table 3.

### 3.2. The Effect of the Process Parameters on the Surface Roughness

The surface roughness of the workpiece was measured using a BRUKER Contour GT-X white light interferometer. (BRUKER GT-X, Billerica, MA, USA). This device was equipped with an inclinometer adjustment stage, allowing for the precise movement of the sample in the X, Y, and Z directions. It also featured an automatic objective/eyepiece changeover system. The base was equipped with an air pressure vibration-damping system, ensuring an excellent stability and vibration resistance. The Vision64 software (2010–2014 Bruker Corporation) was used for the results analysis. The measurement range of the instrument was from 0.01 μm to 250 μm, and the measurement frequency was three measurements per sample, with the average value taken. The center region of the workpiece was selected for measurement to ensure accuracy in the results.

**Figure 4:**
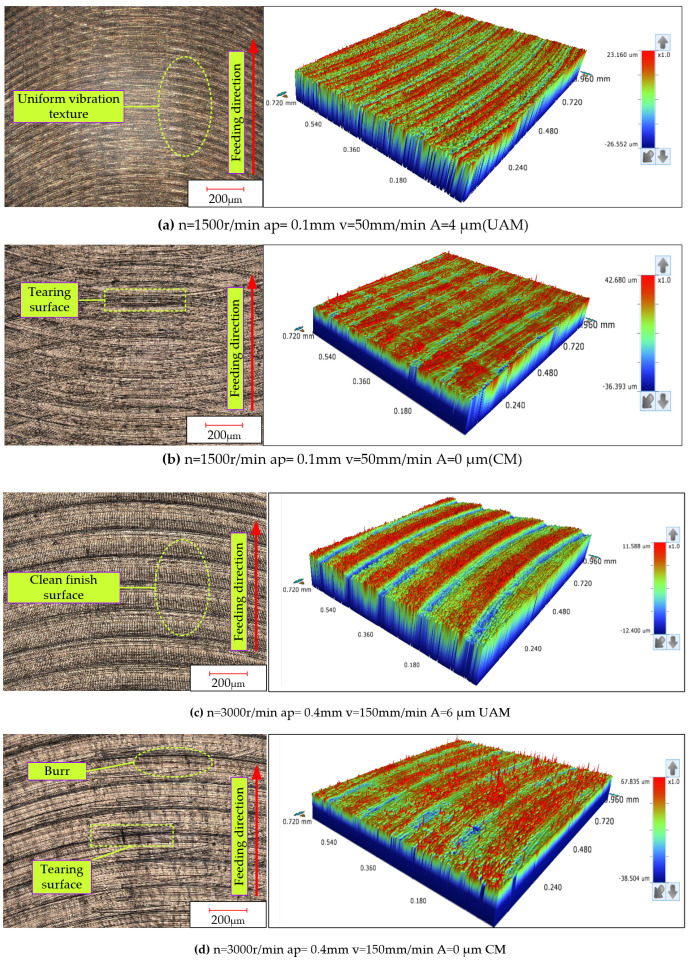

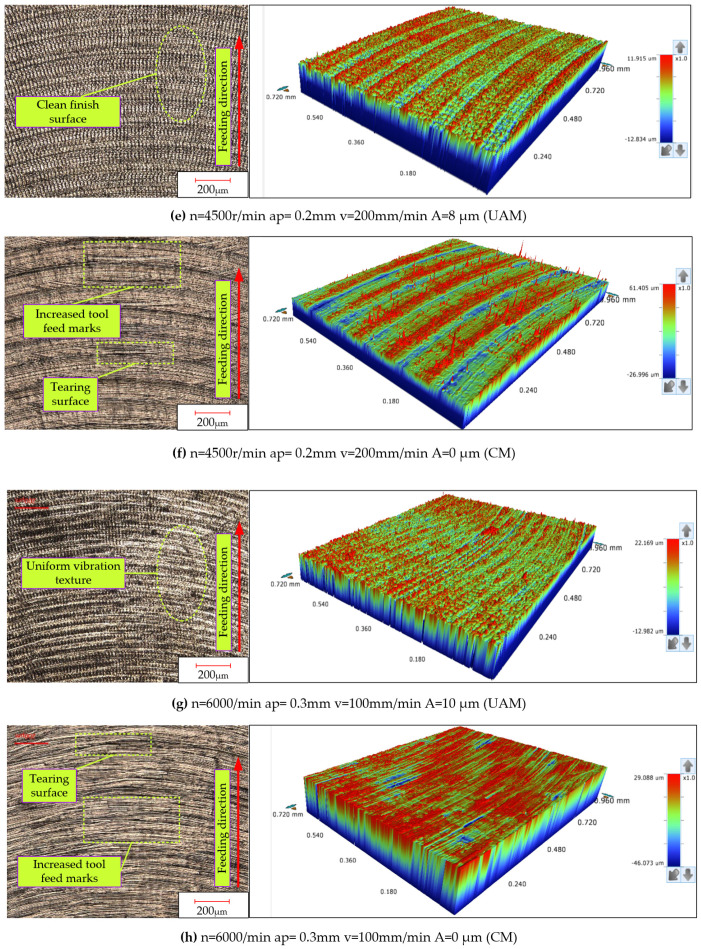
Comparative 3D surface topographies of titanium alloy processed by ultrasonic-assisted milling (UAM) and conventional milling (CM) under varying machining parameters.

Under the same process parameters, as shown in Figure 4, both the ultrasonic-assisted milling and conventional milling experiments were conducted on titanium alloys. Figure 4 shows a comparison of the surface morphology between ultrasonic-vibration-assisted milling and traditional milling under multiple identical process parameters. Under conventional milling conditions, stripe-like features appeared on the machined surface along the direction of tool movement, and material deformation was also very obvious, which made the machined surface rough. During the cutting process, the front cutting surface of the tool exerted pressure on the workpiece material, causing the material to accumulate in front of the tool and flow along the direction of tool movement. However, compared with conventional milling, under ultrasonic-vibration-assisted milling, the surface of the machined workpiece presented a regular, fish-scale-like microscopic texture. Its periodic characteristics stemmed from the impact rebound effect of the high-frequency vibration of the cutting tool on the material. In addition, as the amplitude of ultrasound increased, its effect on surface finishing was further enhanced, and the arrangement of the surface microtextures became more regular.

**Figure 5:**
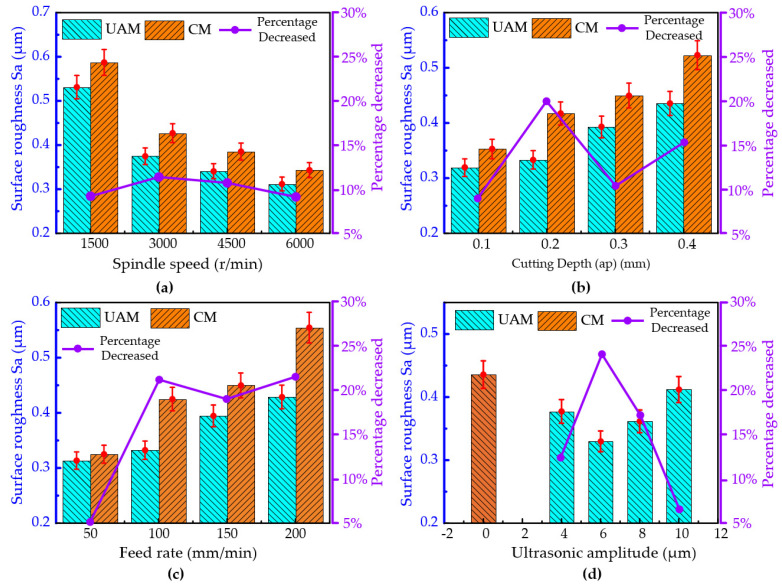
Surface roughness comparison of titanium alloy machined by ultrasonic vibration milling and conventional milling under varying machining parameters. (**a**) Spindle speed, (**b**) Cutting depth, (**c**) Feed rate, and (**d**) Ultrasonic amplitude.

The influence curve of various process parameters on the surface roughness of milled titanium alloys is shown in Figure 5. The process parameters involved included the feed spindle speed *n*, the cutting depth feed rate, and the amplitude.

The influence of different spindle speeds on the surface roughness under conventional milling and ultrasonic-vibration-assisted milling is shown in Figure 5a. It can be seen that, under both cutting methods, as the spindle speed increased, the surface roughness of the machined surface showed a gradually decreasing trend. As the spindle speed increased, the number of times the tool cut the workpiece material per unit time increased, thereby improving the machining quality. Compared with conventional milling, ultrasonic-vibration-assisted milling can significantly improve the machining surface quality, and the surface roughness of the machined surface can be significantly reduced. Moreover, as the spindle speed increased, the improvement effect showed a trend of first increasing and then decreasing. When the spindle speed was 1500 rpm, 3000 rpm, 4500 rpm, and 6000 rpm, the surface roughness decreased by approximately 9.54%, 12.24%, 11.55%, and 9.18%, respectively, under ultrasonic elliptical vibration cutting. When the spindle speed was 3000 r/min, the surface roughness improvement effect was the most significant, with a decrease rate of 12.24%.

Figure 5b illustrates the impact of varying cutting depths on the surface roughness for both conventional milling and ultrasonic-vibration-assisted milling processes. It is apparent that, for both conventional milling or ultrasonic-vibration-assisted milling, with an augmentation in the cutting depth, a significant ascending trend was observed in the surface roughness of the machined surface. However, the amplitude of change for ultrasonic-vibration-assisted milling was relatively small. The change in the cutting depth had a significant impact on the cutting force and the machining quality of the machined surface. As the cutting depth increased, the cutting force gradually increased, the machining difficulty also increased, and the heat accumulation during the cutting process became more obvious, leading to a significant deterioration in the machining quality. Compared with conventional milling, when the cutting depth was set to 0.1 mm, 0.2 mm, 0.3 mm, and 0.4 mm, the surface roughness experienced a reduction of approximately 9.57%, 20.13%, 12.67%, and 16.73%, respectively, under ultrasonic vibration milling conditions.

The influence of different feed rates on the surface roughness under conventional milling and ultrasonic-vibration-assisted milling is shown in Figure 5c. It can be seen that, under both cutting methods, as the feed rate increased, the surface roughness of the machined surface showed an increasing trend. An increase in the feed rate led to an increase in the volume of material that needed to be removed per unit time, resulting in an increase in the cutting force and cutting temperature during the machining process and thereby reducing the machining quality. Compared with conventional milling, the surface roughness under ultrasonic-vibration-assisted milling also showed an increasing trend of change, but it was noticeably smoother than that of conventional milling. When the feed rate was 50 mm/min, the improvement in the surface roughness by ultrasonic-vibration-assisted milling was not significant, about 3%. However, when the feed rate exceeded 50 mm/min, ultrasonic-vibration-assisted milling significantly improved the surface roughness of the machined workpieces. The surface roughness decreased by approximately 3.67%, 21.82%, and 22.68%, corresponding to feed rates of 100 mm/min, 150 mm/min, and 200 mm/min, respectively. The improvement effect of intermittent cutting with periodic contact between the tool and workpiece under ultrasonic-vibration-assisted milling gradually increased with an increase in the feed rate, and eventually tended to flatten out.

Figure 5d illustrates the impact of varying ultrasonic amplitudes on the surface roughness in ultrasonic-vibration-assisted milling processes. Compared to conventional milling, the surface roughness of the machined workpiece was reduced to varying degrees under ultrasonic-vibration-assisted milling. It was evident that, as the ultrasonic amplitude increased from 4 μm to 10 μm, the surface roughness of the machined surface showed a trend of first decreasing and then increasing. In the process of ultrasonic-vibration-assisted milling, when the amplitude varied within the range of 4 μm to 10 μm, its effect on improving the surface roughness of the machined part showed a trend of first increasing and then decreasing. When the ultrasonic amplitude was set to 4 μm, the surface roughness improvement effect was the most significant, with a decrease rate of 24.32%. When the ultrasonic amplitude was set to 10 μm, the improvement effect on the surface roughness was the weakest, with a reduction rate of only 5.44%. This is because the high-frequency impact generated by the ultrasonic amplitude damages the surface quality of the processed material. Therefore, it cannot be simply concluded that a larger ultrasonic amplitude will inevitably lead to a better processing quality. An appropriate ultrasonic amplitude is crucial for improving the processing quality of titanium alloys.

Table 4 shows the range analysis results for the surface roughness (Sa) in the orthogonal experiment. It can be observed that, among the evaluated factors, the spindle speed exhibited the most significant influence on the surface roughness, followed by the cutting depth and the feed rate, while the amplitude had the least impact. k1, k2, k3, and k4 represent the average response values at each factor level, while “r” denotes the response range at each factor level, used to measure the effect size.

### 3.3. The Effect of the Process Parameters on the Cutting Force

**Figure 6:**
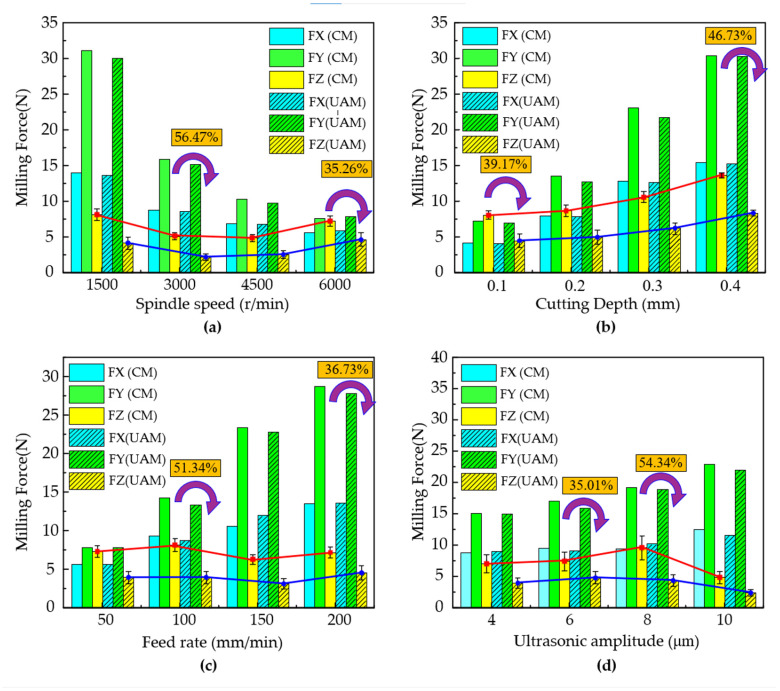
Comparative analysis of directional cutting forces during ultrasonic-assisted milling and conventional milling under various machining parameters. (**a**) Spindle speed, (**b**) Cutting depth, (**c**) Feed rate, and (**d**) Ultrasonic amplitude.

Figure 6 illustrates the impacts of the spindle speed, cutting depth, feed rate, and ultrasonic amplitude on the three-dimensional cutting force. By comparing the three-dimensional cutting force components obtained from conventional milling and ultrasonic-assisted milling, it can be observed that, compared with the cutting forces in the X and Y directions, the reduction in the Z-direction cutting force (Fz) was the most pronounced. Ultrasonic-vibration-assisted milling had a relatively insignificant impact on reducing the cutting forces in the X and Y directions.

As depicted in Figure 6a, the influence of different spindle speeds on the cutting forces in conventional milling and ultrasonic-vibration-assisted milling is presented. Regardless of whether conventional milling or ultrasonic-vibration-assisted milling was used, as the spindle speed increased, the Z-direction cutting force exhibited a trend of first increasing and then decreasing. This is because, as the spindle speed increased, the periodic separation of the tool workpiece caused by ultrasonic vibration gradually deteriorated the cutting heat dissipation effect, leading to an increase in the cutting force of ultrasonic-vibration-assisted milling. Compared with conventional milling, ultrasonic-vibration-assisted milling achieved a reduction ratio range of cutting forces from 35.26% to 56.47%, with the maximum reduction occurring at a spindle speed of 3000 rpm.

As shown in Figure 6b, the influence of different cutting depths on the cutting forces in conventional milling and ultrasonic-vibration-assisted milling is presented. It can be clearly observed that, regardless of whether conventional milling or ultrasonic-vibration-assisted milling was used, as the cutting depth increased, the three-directional cutting forces exhibited a significant increasing trend. Compared with conventional milling, ultrasonic-vibration-assisted milling demonstrated a more pronounced reduction in the Z-direction cutting force, and this reduction magnitude increased with the augmentation of the cutting depth. Specifically, when the cutting depth was 0.1 mm, the minimum percentage reduction in the Z-direction cutting force achieved by ultrasonic-vibration-assisted milling was 39.17%; when the cutting depth reached 0.4 mm, the maximum percentage reduction was 46.73%.

As illustrated in Figure 6c, the influence of different feed rates on the cutting forces in conventional milling and ultrasonic-vibration-assisted milling is presented. It can be clearly observed from the figure that, regardless of whether conventional milling or ultrasonic-vibration-assisted milling was used, as the feed rate increased, the cutting forces in the X and Y directions exhibited a significant increasing trend. However, under both cutting methods, the variation in the Z-direction cutting force with the feed rate was not remarkable and tended to remain stable. Compared with conventional cutting, ultrasonic-vibration-assisted cutting can significantly reduce the Z-direction cutting force. When the feed rate was 100 mm/min, the maximum percentage reduction in the Z-direction cutting force achieved by ultrasonic-vibration-assisted milling was 51.34%; when the feed rate reached 0.4 mm, the minimum percentage reduction was 36.73%.

The variation in the cutting force under different ultrasonic amplitudes is shown in Figure 6d. The ultrasonic amplitude was set to 4 μm, 6 μm, 8 μm, or 10 μm. The results showed that, when the feed rate was increased from 50 mm/min to 150 mm/min, the cutting temperature of the milling area of the titanium alloy first decreased and then increased with the increase in feed rate. When the feed rate exceeded 150 mm/min, the cutting temperature tended to stabilize. Similarly, at different feed rates, the cutting temperature in ultrasonic-assisted milling was lower than that of conventional milling. The reason for this phenomenon is that the high-frequency reciprocating motion of the tool reduced the contact area between the chip and the rake face of the tool, thereby effectively decreasing the friction power consumption.

Table 5 presents the analysis outcomes regarding the range of the Z-direction cutting force under various process parameters in orthogonal experiments. From the table, it is evident that, among the factors influencing the Z-direction cutting force, the cutting depth exerted the most pronounced effect. This was followed by the spindle speed and the ultrasonic amplitude. In contrast, the feed rate had the least influence on the cutting temperature.

### 3.4. The Effect of the Process Parameters on the Cutting Temperature

**Figure 7:**
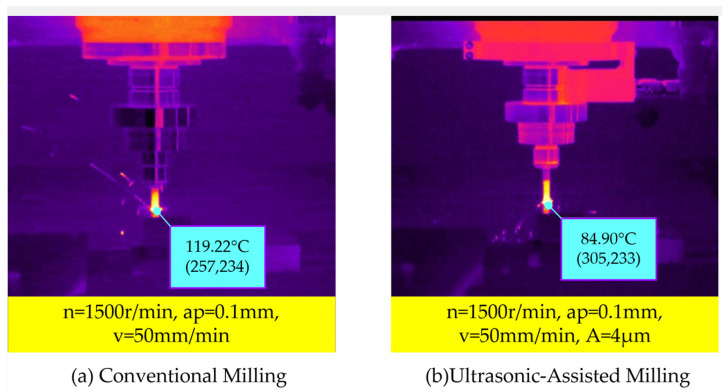
Comparison of cutting temperature between CM and UAM.

A comparison of the cutting temperature of titanium alloys for both conventional milling and ultrasonic-assisted milling under the same cutting parameters is shown in Figure 7. Significant differences in the cutting temperature were observed between the two cutting methods. The temperature of the ultrasonic tool holder in ultrasonic-assisted milling was much higher than that in conventional milling, due to the high-frequency heating phenomenon of piezoelectric actuators. However, compared with the cutting temperature of 119.22 °C in conventional milling, the cutting temperature was 84.90 °C in ultrasonic-assisted cutting—the cutting temperature decreased by 28.8%.

**Figure 8:**
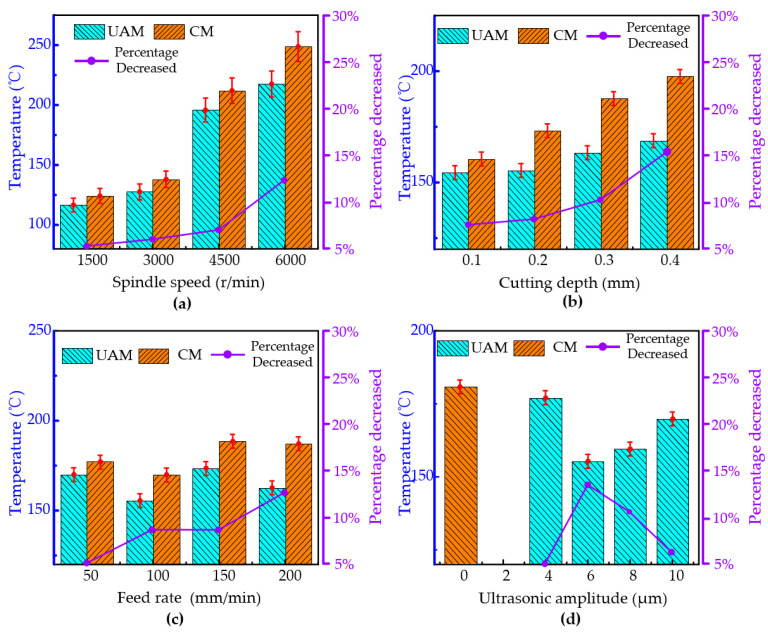
Cutting temperature comparison of titanium alloy machined by ultrasonic vibration milling and conventional milling under varying machining parameters. (**a**) Spindle speed, (**b**) Cutting depth, (**c**) Feed rate, and (**d**) Ultrasonic amplitude.

The effects of the spindle speed, cutting depth, feed rate, and ultrasonic amplitude on the cutting temperature are shown in Figure 8.

The influence of the spindle speed on the cutting temperature is illustrated in Figure 8a. When the spindle speed increased from 1500 r/min to 6000 r/min, the cutting temperature rose significantly for both conventional milling and ultrasonic-vibration-assisted milling. However, the cutting temperature for ultrasonic-vibration-assisted milling was lower than that for traditional milling at the same spindle speed. Compared to conventional milling, at a spindle speed of 1500 rpm, 3000 rpm, 4500 rpm, and 6000 rpm, the cutting temperature decreased by approximately 5.12%, 6.32%, 7.22%, and 12.57%, respectively, under ultrasonic elliptical vibration cutting. The reason for this phenomenon is that, during the ultrasonic vibration milling process, the intermittent contact between the tool and the workpiece reduces the actual cutting time and cutting force, resulting in a decrease in the cutting temperature.

As shown in Figure 8b, different cutting depths have a notable impact on the cutting temperatures for both conventional milling and ultrasonic-vibration-assisted milling. The results indicate that, regardless of whether conventional milling or ultrasonic-vibration-assisted milling was used, the cutting temperature gradually increased with the rising cutting depth. Compared to conventional milling, ultrasonic-vibration-assisted milling exhibited lower cutting temperatures. Specifically, when the cutting depths were 0.1 mm, 0.2 mm, 0.3 mm, and 0.4 mm, the reduction percentages of the cutting temperature in ultrasonic-vibration-assisted milling reached 8.34%, 9.13%, 10.96%, and 15.18%, respectively, showing a gradually increasing trend.

The variation in the cutting temperature at different cutting depths is shown in Figure 8c. The feed rate was set to 50 mm/min, 100 mm/min, 150 mm/min, or 200 mm/min. The results showed that, when the feed rate was increased from 50 mm/min to 150 mm/min, the cutting temperature of the milling area of the titanium alloy first decreased and then increased with the increase in feed rate. When the feed rate exceeded 150 mm/min, the cutting temperature tended to stabilize. Similarly, at different feed rates, the cutting temperature in ultrasonic-assisted milling was lower than that of conventional milling. The reason for this phenomenon is that the high-frequency reciprocating motion of the tool reduces the contact area between the chip and the rake face of the tool, thereby effectively decreasing the friction power consumption.

The cutting temperature was measured at different ultrasonic amplitudes, and the experimental data were used to plot the variation in the cutting temperature with ultrasonic amplitude, as shown in Figure 8d. The ultrasonic amplitude was set to 4 µm, 6 µm, 8 µm, or 10 µm. The results showed that, when the feed rate was increased from 4 µm to 10 µm, the cutting temperature under ultrasonic-assisted milling initially decreased and then increased with the increase in the ultrasonic amplitude. Compared to conventional milling, when the ultrasonic amplitude was 6 µm, the maximum percentage decrease in the cutting temperature of 14.05% was achieved, while when the ultrasonic amplitude was 4 µm, the minimum percentage decrease in the cutting temperature of 2.06% was observed.

Table 6 shows the analysis results of the cutting temperature range under different process parameters in orthogonal experiments. As illustrated in Table 6, it can be seen that, among the factors affecting cutting temperature, the spindle speed had the most significant impact on the cutting temperature, followed by the cutting depth and amplitude, while the feed rate had the smallest impact on the cutting temperature.

## 4. Grey Correlation Analysis

### 4.1. Data Standardization Processing

During the milling process of titanium alloys, the surface roughness Sa, cutting temperature, and cutting forces FX, FY, and FZ are considered “look-to-minimize” characteristics. This implies that the optimization objective for these characteristics is to minimize their values as much as possible, so as to achieve better surface quality. To ensure that these characteristics have a unified dimension, it is often necessary to standardize the original data [28]. Generally, the standardization process is carried out using the following Formula (4):

(4) $$x_{i}^{\;*}(k)=\frac{x_{i}(k)-min(x_{i})}{max(x_{i})-min(x_{i})}$$

In this equation, xi*(k) represents the comparison sequence after standardization and xi(k) represents the original data sequence. i denotes the test number, where i = 1,2,3……, 16, and k represents the evaluation criteria, with k = 1, 2, 3, 4, 5. max(xi) and min(xi) represent the maximum and minimum values in the original data, respectively.

According to the formula and normalization process, the corresponding normalized data sequence is shown in Table 7.

### 4.2. Grey Correlation Coefficient and Grey Correlation Degree

The grey relational coefficient is used to represent the degree of association between a reference sequence and a comparison sequence at a given point in time [29]. Its value ranges between 0 and 1. The formula for calculating the grey relational coefficient is as follows:

(5) $$\delta_{i}(k)=\frac{min_{\forall i}min_{\forall k}\Delta_{i}(k)+\xi max_{\forall i}max_{\forall k}\Delta_{i}(k)}{\Delta_{i}(k)+\xi max_{\forall i}max_{\forall k}\Delta_{i}(k)}$$

Δi(k) represents the difference between the i-th comparison sequence and the reference sequence at time k. Δi(k) can be calculated as Δi(k) = |x0*(k) − xi*(k)|, where x0*(k) is the reference sequence with a value of 1.0. Assume that Δmin and Δmin are the minimum and maximum values among all the difference sequences, respectively. ξ is the distinguishing coefficient, typically set to 0.5. The corresponding difference sequence is shown in Table 8.

The grey relational coefficient is used to measure the similarity between the reference and comparison sequences at a specific time point [30]. A value closer to 1 indicates a stronger correlation, while a value closer to 0 indicates a weaker one. The average of the grey relational coefficients across all the evaluation criteria is defined as the grey relational grade, calculated as shown in Equation (6).

(6) $$\delta_{k}=\frac{1}{m}\sum_{i=1}^{m}\xi_{i}(k)$$

In this equation, m denotes the number of evaluation criteria, with m = 5. The calculated grey relational degrees are shown in Table 9. The grey relational degree reflects the level of association between the reference sequence and each comparison sequence. A higher value indicates a closer proximity to the optimal target. Without considering the specific types of process parameters, a larger grey relational degree implies better performance of the corresponding parameter combination. As shown in Table 9, the highest grey relational degree was 0.869, corresponding to the 13th set of process parameters: a spindle speed of 6000 r/min, a feed rate of 200 mm/min, an ultrasonic amplitude of 6 μm, and a cutting depth of 0.2 mm.

### 4.3. Grey Relational Coefficient and Grey Relational Grade

**Figure 9:**
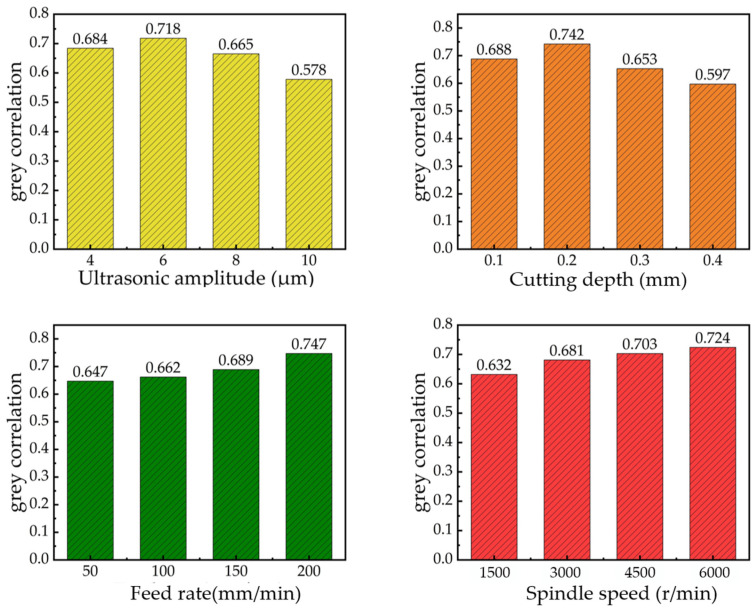
Horizontal average grey correlation of process parameters.

Figure 9 illustrates the average grey relational grade under different parameter levels. As shown in the figure, when the surface roughness, cutting force, and cutting temperature are considered as optimization objectives, the optimal process parameter combination for the ultrasonic vibration milling of TC4 titanium alloys is a spindle speed of 6000 r/min, a feed rate of 200 mm/min, an ultrasonic amplitude of 6 μm, and a cutting depth of 0.2 mm.

### 4.4. Experimental Validation

Through a grey relational analysis, the optimal parameter combination for the ultrasonic vibration milling of titanium alloys was determined to be a spindle speed of 6000 r/min, a feed rate of 200 mm/min, an ultrasonic amplitude of 6 μm, and a cutting depth of 0.2 mm. Ultrasonic milling experiments were conducted using this parameter set, and the results shown in Table 10 indicate a grey relational grade of 0.873, which exceeds the highest value of 0.869 observed in the 13th group of the orthogonal tests. This demonstrates that the optimized parameters can effectively improve surface roughness, cutting force, and cutting temperature in the machining of titanium alloys. The appropriate process parameters can be selected by utilizing the optimization method proposed in this article according to different working conditions, which has important engineering value for the high-quality processing of titanium alloys.

## 5. Conclusions

This study investigated how various process parameters influence the surface roughness, cutting force, and cutting temperature during the ultrasonic vibration milling of TC4 titanium alloys, using data from orthogonal experiments. Additionally, multi-objective parameter optimization was performed for the ultrasonic vibration milling of TC4 titanium alloys, leading to the following conclusions:

The influence of different process parameters on the surface roughness can be ranked as follows: the spindle speed has the most significant effect, followed by the cutting depth and feed rate, while the ultrasonic amplitude has the least impact. Regarding the cutting force, compared to conventional milling, ultrasonic vibration milling leads to a more considerable reduction in the Z-direction force (FZ), with relatively less effect on the X- and Y-direction forces. Among these factors, the cutting depth has the most substantial effect on the Z-direction cutting force, followed by the spindle speed and the ultrasonic amplitude. The spindle speed has the least influence on the cutting force. As for the cutting temperature, the spindle speed has the most significant impact, followed by the cutting depth and the ultrasonic amplitude, while the feed rate shows the least sensitivity to temperature variations.

Using the grey correlation analysis method, with the surface roughness, cutting force, and cutting temperature as the objectives, the optimal process parameters identified were as follows: a spindle speed of 6000 rpm, a cutting depth of 0.2 mm, a feed rate of 200 mm/min, and an ultrasonic amplitude of 6 μm. The experimental results showed that the surface roughness (Sa) was 0.268 μm, the cutting temperature was 255.39 °C, and the cutting force in the X direction (FX) was 5.2 N, in the Y direction (FY) was 7.9 N, and in the Z direction (FZ) was 6.4 N. Optimizing the process parameters can significantly improve the machining quality of titanium alloys and reduce cutting forces and temperatures, and is highly significant for the high-quality machining of titanium alloys.

## Figures and Tables

**Table 1:** Main chemical components of TC4 titanium alloy.

Element	Ti	Al	V	Fe	C	N	H	O
*Wt* (%)	51.75	17	5.15	2.93	1.07	0.45	0.042	0.21

**Table 2:** Main mechanical properties of TC4 titanium alloy.

Property	Elastic Modulus (GPa)	Poisson’s Ratio	Hardness (HV)	Yield Strength (MPa)	Tensile Strength (MPa)	Elongation (%)
TC4	110	0.34	310	830	895	10

**Table 3:** Orthogonal test factors and levels.

Level	Spindle Speed *n* (r/min)	Cutting Depth ap (mm)	Feed Rate vf (mm/min)	Amplitude A (μm)
1	1500	0.1	50	4
2	3000	0.2	100	6
3	4500	0.3	150	8
4	6000	0.4	200	10

**Table 4:** Range analysis results of surface roughness from orthogonal experiment.

No.	Experimental Parameters			
	Spindle Speed *n* (r/min)	Cutting Depth ap (mm)	Feed Rate vf mm/min	Amplitude A (μm)
k1	0.48	0.32	0.32	0.38
k2	0.37	0.33	0.33	0.33
k3	0.32	0.39	0.43	0.36
k4	0.31	0.44	0.39	0.41
r	−0.18	−0.12	−0.1	−0.08

**Table 5:** Analysis results of Z-direction cutting force range in orthogonal experiment.

No.	Experimental Parameters			
	Spindle Speed *n* (r/min)	Cutting Depth ap (mm)	Feed Rate vf mm/min	Amplitude A (μm)
k1	4.777	4.518	3.963	3.974
k2	2.559	5.012	3.981	4.842
k3	2.969	4.103	3.149	4.391
k4	5.350	2.022	4.562	2.449
r	2.791	2.990	1.413	2.393

**Table 6:** Analysis results of temperature range in orthogonal experimental cutting.

No.	Experimental Parameters			
	Spindle Speed *n* (r/min)	Cutting Depth ap (mm)	Feed Rate vf mm/min	Amplitude A (μm)
k1	113.36	159.6	170.01	169.82
k2	132.03	193.07	155.54	177.03
k3	198.83	145.86	173.5	155.34
k4	217.5	163.18	162.66	159.52
r	−104.14	−47.21	−17.97	−21.68

**Table 7:** Comparison sequence.

Serial Number	Comparison Sequence				
	Sa	Temperature	FX	FY	FZ
1	0.73512	1.00000	0.95634	0.96269	0.42962
2	0.61905	0.83564	0.70895	0.75363	0.00000
3	0.00000	0.84609	0.29172	0.35213	0.44076
4	0.10119	0.71560	0.00000	0.00000	0.80306
5	0.85417	0.72661	0.97404	0.98024	0.60245
6	0.91369	0.50400	0.94585	0.99563	0.73892
7	0.67560	0.83442	0.55698	0.66132	0.41787
8	0.30952	0.08090	0.46189	0.58507	1.00000
9	0.82738	0.43836	0.96011	0.98141	0.67650
10	0.74107	0.55731	0.74670	0.85084	0.46610
11	0.98214	0.42848	0.87610	0.98105	0.55006
12	0.90476	0.36458	0.69129	0.82824	0.86585
13	1.00000	0.36208	1.00000	1.00000	0.09155
14	0.97321	0.00000	0.86041	0.92942	0.35337
15	0.88095	0.67664	0.73822	0.92732	0.59486
16	0.64881	0.39855	0.86041	0.92942	0.35337

**Table 8:** Difference sequences.

Serial Number	Difference Series				
	Sa	Temperature	FX	FY	FZ
1	0.265	0.000	0.042	0.037	0.570
2	0.309	0.167	0.207	0.234	0.000
3	0.000	0.153	0.000	0.000	0.439
4	0.051	0.285	0.000	0.000	0.803
5	0.427	0.274	0.286	0.302	0.600
6	0.456	0.500	0.268	0.324	0.737
7	0.338	0.166	0.171	0.160	0.417
8	0.155	0.721	0.113	0.138	0.997
9	0.413	0.000	0.303	0.308	0.674
10	0.371	0.400	0.218	0.249	0.465
11	0.492	0.000	0.244	0.243	0.549
12	0.453	0.000	0.183	0.172	0.863
13	0.505	0.000	0.000	0.000	0.091
14	0.486	0.000	0.251	0.245	0.354
15	0.440	0.326	0.228	0.244	0.594
16	0.324	0.000	0.251	0.245	0.354

**Table 9:** Grey relational coefficients.

Serial Number	Grey Correlation Coefficient						
	Sa	Temp	FX	FY	FZ	Relatedness	Rank
1	0.653	1.000	0.922	0.931	0.467	0.795	3
2	0.617	0.749	0.707	0.681	1.000	0.751	5
3	1.000	0.765	1.000	1.000	0.531	0.859	2
4	0.907	0.636	1.000	1.000	0.383	0.785	4
5	0.539	0.645	0.635	0.623	0.454	0.579	15
6	0.522	0.499	0.65	0.606	0.404	0.536	16
7	0.596	0.75	0.745	0.757	0.544	0.678	8
8	0.763	0.409	0.815	0.783	0.333	0.621	12
9	0.547	1.000	0.622	0.618	0.425	0.642	11
10	0.573	0.555	0.696	0.667	0.518	0.602	13
11	0.503	1.000	0.671	0.672	0.476	0.664	10
12	0.524	1.000	0.731	0.744	0.366	0.673	8
13	0.497	1.000	1.000	1.000	0.846	0.869	1
14	0.506	1.000	0.665	0.67	0.585	0.685	7
15	0.531	0.605	0.686	0.671	0.456	0.590	14
16	0.606	1.000	0.665	0.670	0.585	0.705	6

**Table 10:** Test validation results.

Spindle Speed *n* (r/min)	Cutting Depth ap (mm)	Feed Rate vf (mm/min)	Amplitude A (μm)	Surface Roughness Sa(μm)	Cutting Temperature (°C)	FX (N)	FY (N)	FZ (N)
6000	0.2	200	6	0.268	255.39	5.2	7.9	6.4

## Data Availability

The original contributions presented in this study are included in the article; further inquiries can be directed to the corresponding author.
